# Loss of Suppressor of Fused in Mid-Corticogenesis Leads to the Expansion of Intermediate Progenitors

**DOI:** 10.3390/jdb4040029

**Published:** 2016-09-29

**Authors:** Odessa R. Yabut, Hui Xuan Ng, Gloria Fernandez, Keejung Yoon, Jeremy Kuhn, Samuel J. Pleasure

**Affiliations:** 1 Department of Neurology, University of California San Francisco, San Francisco, CA 94158, USA; odessa.yabut@ucsf.edu (O.R.Y.); huixuan.ng@ucsf.edu (H.X.N.); gloria__fg@hotmail.com (G.F.); keejung@skku.edu (K.Y.); jeremydavidkuhn@gmail.com (J.K.); 2 College of Biotechnology and Bioengineering, Sungkyunkwan University, Suwon, Gyeonggi-do 16419, Korea; 3 Programs in Neuroscience and Developmental Biology, Eli and Edythe Broad Center of Regeneration Medicine and Stem Cell Research, University of California San Francisco, San Francisco, CA 94143, USA

**Keywords:** Suppressor of Fused, Sonic Hedgehog, Gli3, corticogenesis, neurogenesis

## Abstract

Neural progenitors in the embryonic neocortex must be tightly regulated in order to generate the correct number and projection neuron subtypes necessary for the formation of functional neocortical circuits. In this study, we show that the intracellular protein Suppressor of Fused (Sufu) regulates the proliferation of intermediate progenitor (IP) cells at later stages of corticogenesis to affect the number of Cux1+ upper layer neurons in the postnatal neocortex. This correlates with abnormal levels of the repressor form of Gli3 (Gli3R) and the ectopic expression of Patched 1 (Ptch1), a Sonic Hedgehog (Shh) target gene. These studies reveal that the canonical role of Sufu as an inhibitor of Shh signaling is conserved at later stages of corticogenesis and that Sufu plays a crucial role in regulating neuronal number by controlling the cell cycle dynamics of IP cells in the embryonic neocortex.

## 1. Introduction

The generation of molecularly diverse glutamatergic projection neurons in the adult mammalian neocortex is dynamically regulated by spatial and temporal events during embryonic cortical development. Virtually all glutamatergic projection neurons originate from neural progenitors in the ventricular (VZ) and subventricular zone (SVZ), which are derived from multipotent radial glial (RG) cells [1]. At early stages of corticogenesis, the majority of RGs initially expand through self-renewing symmetric divisions at the apical surface of the VZ. As corticogenesis progresses, RG cells undergo asymmetric neurogenic divisions to self-renew and generate intermediate progenitors (IP) or short neural precursors (SNP) [2,3]. IP cells that populate the SVZ at later stages of corticogenesis will undergo one or more symmetric divisions before terminally differentiating to produce a pair of (or four) neurons that will largely populate the upper layers of the neocortex [4]. This temporal sequence is a fundamental feature of neocortical development, which ensures the generation of correct number of projection neurons, necessary for precise formation of local and long-range circuits in the adult neocortex.

Specialized molecular cascades incorporate intrinsic and extrinsic cues to direct the expansion and differentiation of neural progenitors in the telencephalon. Sonic Hedgehog (Shh) signaling is centrally involved in this process and is required during distinct developmental windows to direct patterning, specification, and proliferation of progenitors [5]. In the dorsal telencephalon, Shh signaling activity is relatively low during corticogenesis, yet deregulation of Shh signaling in the embryonic neocortex profoundly affects the behavior of neural stem/progenitor cells. In particular, we recently found that when modulation of Shh signaling is lost at early stages of corticogenesis, neocortical progenitors are improperly specified, which results in the misspecification of projection neurons [6]. Tightly regulating Shh signaling is also likely crucial in late corticogenesis, since loss of transcription repressor activity of Gli3, a downstream effector of Shh signaling, leads to abnormal proliferation and specification of cortical progenitors [7]. Similarly, forced overexpression of Shh at this stage results in the abnormal specification of cortical progenitors [8]. However, the implications of deregulating endogenous Shh signaling in late corticogenesis have not been yet fully examined.

Suppressor of Fused (Sufu) is a critical antagonist of Shh signaling that influences patterning, specification, differentiation, and migration of neural progenitors and their progenies in the developing central nervous system [6,9,10,11]. Sufu exerts this role by directly acting on Gli transcription factors downstream of the Shh signaling cascade via control of their stability, degradation, and the formation of Gli repressors [11,12,13,14]. At early stages of corticogenesis, we have shown that Sufu modulates Shh signaling by controlling the stability of full-length Gli2 and Gli3 [6]. These defects were absent at later stages of corticogenesis, where Gli3, the predominant Gli at this stage, was not similarly affected and all projection neurons of the neocortex were properly specified. However, in-depth studies on the state of cortical progenitors at later stages of corticogenesis and the effect of endogenous Shh signaling are needed, particularly since additional Shh-expressing cells are present in a subset of progenitors lining the lateral ventricles, Cajal-Retzius neurons, and in ventrally derived interneurons that have migrated into the neocortex [15]. Furthermore, loss of function studies indicate that Shh signaling continues to play a role in regulating the cell cycle dynamics of cortical progenitors [15]. In this study, we show that Sufu continues to function as a regulator of Shh signaling activity at later stages of corticogenesis. Specifically, loss of Sufu deregulates the proliferation program of basally dividing cortical progenitors resulting in an increase in the number of upper layer neurons in the postnatal neocortex. These observations correlate with an increase in Shh activity, in part due to an imbalance between the full-length Gli3 activator (Gli3FL) and the cleaved Gli3R repressor (Gli3R) levels. These studies elucidate an important role for Sufu in regulating Shh signaling activity in late corticogenesis.

## 2. Materials and Methods

### 2.1. Animals

Mice carrying the floxed Sufu allele (*Sufu^fl^*) were kindly provided by Dr. Chi-Chung Hui (University of Toronto, Toronto, ON, Canada) and were genotyped as described [16]. The *hGFAP-Cre* (Stock #004600) was obtained from Jackson Laboratories (Bar Harbor, ME, USA). All animal protocols were in accordance with the regulations of the National Institute of Health and approved by the University of California San Francisco Institutional Animal Care and Use Committee (IACUC).

### 2.2. Immunohistochemistry and DiI Labeling

Perfusion, dissection, and immunofluorescence staining were conducted according to standard protocols as previously described [17]. The following are the antibodies used: mouse anti-BrdU (1:50 dilution; BD Pharmingen, Franklin Lakes, NJ, USA), rabbit anti-Cux1 (1:100 dilution; Santa Cruz Biotechnology, Dallas, TX, USA), rabbit anti-Phospho-Histone H3 (1:250 dilution; Millipore, Billerica, MA, USA), rabbit anti-Pax 6 (1:500 dilution; Covance, Princeton, NJ, USA), rabbit anti-Tbr2 (1:500 dilution; Abcam, Cambridge, UK), mouse anti-Tuj1 (1:500 dilution; Covance, Princeton, NJ, USA), rabbit anti-Gli3 (1:100 dilution; Santa Cruz Biotechnology, Dallas, TX, USA), and rabbit anti-Cleaved Caspase 3 (1:300 dilution; Cell Signaling, Madison, WI, USA).

For 5-bromo-2-deoxyuridine (BrdU, Sigma, St. Louis, MO, USA) labeling, pregnant dams were treated with 50 μg/g BrdU by intraperitoneal injection for 4 h prior to dissection at E16.5. DiI labeling was conducted by placing small crystals of the lipophilic tracer (1,1′-dioctadecyl-3,3,3′,3′-tetramethylindocarbocyanine; Invitrogen, Waltham, MA, USA) in the neocortex to target the upper layer (2/3) and then remained in 4% paraformaldehyde (PFA). After 6 weeks, brains were sectioned at 100 μm, counterstained with bisbenzimide, mounted, and imaged.

### 2.3. In Situ Hybridization

Patched 1 (Ptch1) in situ hybridization (ISH) was conducted according to standard protocols [17] using probes generated from mouse ptc probe M2-3, a gift from Matthew Scott (Addgene plasmid #58701).

### 2.4. Image Acquisition and Analysis

Images were acquired using a Nikon E600 microscope equipped with a QCapture Pro camera (QImaging) or Zeiss Axioscan Z.1 (Zeiss, Thornwood, NY, USA) using the Zen 2 blue edition software (Zeiss, Thornwood, NY, USA. Z-stack images were acquired using a Nikon Spectral C1si Laser Scanning Confocal (Nikon Imaging Center, UCSF) and scanned a 10 μm section at a resolution of 1024 × 1024 pixels using the Nikon EZ-C1 software v3.8 (Nikon Instruments, Melville, NY, USA). Adobe Photoshop CS6 was used for image editing with brightness/contrast levels adjusted equally in control and mutant tissues. NIH Image J was used to quantify raw, unedited images. For measurement of VZ/SVZ thickness, the length of densely populated 4′,6-diamidino-2-phenylindole (DAPI)+ regions adjacent to the lateral ventricles to the SVZ/intermediate zone (IZ) boundary was measured. For measurement of BrdU-labeled cells in the VZ or SVZ, the VZ was defined as the area within 70 μm from the lateral ventricles, while the SVZ was defined as the rest of the DAPI-dense regions along the progenitor zones. Pax6+, Tbr2+, and BrdU+, and phospho-Histone H3+ cells were quantified within the VZ/SVZ, which was characterized by the densely populated DAPI+ regions adjacent to the lateral ventricles or by where Tuj1 staining was sparse. Fluorescently labeled cells within this region were quantified to measure the number of cells per 100 μm^2^. Apical progenitors were quantified by measuring the number of labeled cells along the length of the ventricular surface. For quantification of labeled Cux1+ neurons at postnatal stages, layers 2/3/4 were measured to determine the area. Fluorescently labeled cells within this region were quantified to measure the number of cells per 100 μm^2^. Two to three optical sections at 10 μm-thick each, which were histologically matched for rostral-caudal level between genotypes, were analyzed.

### 2.5. Quantitative PCR Analysis

Total RNA was isolated from dissected E16.5 cortical tissues using the RNEasy Mini Kit (Qiagen, Venlo, The Netherlands). cDNA was generated using the High Capacity cDNA Reverse Transcription Kit (Applied Biosystems, Waltham, MA, USA). Transcript expression was measured via the incorporation of SYBR Green (Life Technologies, Carlsbad, CA, USA) using the Applied Biosystem 7500 Real-Time PCR System (Life Technologies, Carlsbad, CA, USA). Primers for Ptch1 and Gli1 have been previously described [18]. Quantitative PCR (qPCR) data were analyzed using the comparative C_T_ or the relative standard curve method, with β-actin [19] used as control.

### 2.6. Western Blot Analysis

Cortical tissues were lysed in radioimmunoprecipitation assay (RIPA) buffer (Sigma) supplemented with protease (Complete Mini, Roche, Basel, Switzerland) and phosphatase (PhosStop, Roche) inhibitors according to standard protocols. Western blot analyses were conducted according to standard protocols. Briefly, soluble extracts were loaded onto Criterion, 4%–15% Tris-HCI 4 sodium dodecyl sulfate polyacrylamide gel electrophoresis (SDS-PAGE) gels (Bio-Rad, Berkeley, CA, USA), separated at 120 V, and transferred to polyvinylidene difluoride (PVDF) membrane (Bio-Rad) at 30 V for 2 h or overnight at 4 °C. Membranes were blocked with 5% BSA/1X TBS-T (Tris-buffered saline with 0.1% Tween 20) for 1 h at room temperature, and incubated with primary antibodies diluted in blocking buffer overnight at 4 °C, and secondary antibodies (1:5000 dilution; IR-Dye antibodies, LI-COR, Lincoln, NE, USA) for 1 h at RT. Membranes were washed in 1X TBS-T and scanned using the Odyssey Infrared Imaging System (LI-COR). Primary antibodies were used as follows: goat anti-Gli3 (1:500; R&D Systems) and α-Tubulin (1:5000, Abcam). Quantification and analysis were conducted using the Odyssey System and Image Studio Software version 4.0.21 (LI-COR, Lincoln, NE, USA).

### 2.7. Statistics

All experiments were conducted in triplicate with a sample size of *n* = 3−6 embryos/animals per genotype. For pairwise analysis of control and mutant genotypes, the Student t-test was used. Graphs display the mean ± standard error of the mean (SEM).

## 3. Results

### 3.1. Expansion of the VZ and SVZ in the E16.5 Neocortex of hGFAP^cre/+^;Sufu^fl/fl^ Mice

**Figure 1:**
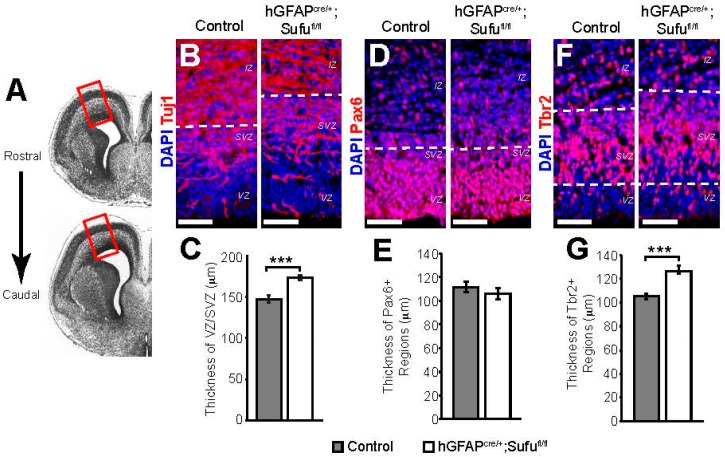
Expansion of the progenitor zones in E16.5 *hGFAP^cre/+^;Sufu^fl/fl^* mice. (**A**) The experiments presented here analyzed the dorsolateral region of the neocortex (boxed region), obtained from the rostral forebrain spanning between these representative images; (**B**,**C**) Targeted deletion of Suppressor of Fused (Sufu) in cortical progenitors at E13.5 (*hGFAP^cre/+^;Sufu^fl/fl^*) led to the expansion of the VZ/SVZ by E16.5 as defined by immunofluorescence staining. Regions marked by DAPI-dense (blue) cells and reduced Tuj1 (red) immunostaining along the lateral ventricles showed expansion of the *hGFAP^cre/+^;Sufu^fl/fl^* VZ/SVZ compared to controls (**B**); The thickness of the VZ/SVZ was determined as the distance between the ventricular lining along the lateral ventricles and the IZ, which was identified by densely packed DAPI+ cells and minimal Tuj1 staining within the dorsolateral neocortex. These measurements confirmed that the *hGFAP^cre/+^;Sufu^fl/fl^* VZ/SVZ was significantly thicker than those of control littermates (**C**); (**D**,**E**) Immunofluorescence staining against the RG cell marker, Pax6 (red), showed no visible differences between the E16.5 control and *hGFAP^cre/+^;Sufu^fl/fl^* Pax6+ regions as defined by the dashed lines (**D**); This observation was verified when the thickness of Pax6+ regions was measured (**E**); (**F**,**G**) Immunofluorescence staining against the intermediate progenitor (IP) cell marker, Tbr2 (red), showed a visible expansion of Tbr2+ regions (between dashed lines) in the E16.5 *hGFAP^cre/+^;Sufu^fl/fl^* VZ/SVZ when compared to controls (**F**); Quantification of the thickness of Tbr2+ regions verified these observations (**G**); *** *p*-value < 0.01. Scale bars = 50 μm; VZ, ventricular zone; SVZ, subventricular zone; IZ, intermediate zone; DAPI, 4′,6-diamidino-2-phenylindole.

We previously determined that Sufu has critical functions at early stages of corticogenesis particularly in the specification of cortical progenitors into distinct neuronal lineages [6]. Since Sufu remains specifically expressed in the VZ/SVZ of the embryonic neocortex mid-corticogenesis [6], we postulated that it continues to play important roles in cortical progenitors at this stage. To identify the function of Sufu, we examined how loss of Sufu affected the behavior of cortical progenitors within the dorsolateral regions of the rostral neocortex (Figure 1A) in *hGFAP^cre/+^;Sufu^fl/fl^* mice, in which Sufu deletion in all cortical progenitors occurred mid-corticogenesis (~E13.5) [20]. In *hGFAP^cre/+^;Sufu^fl/fl^* mice, the specification of cortical progenitors was relatively normal [6]. However, we observed an expansion of the VZ/SVZ at E16.5 in the dorsolateral neocortex of *hGFAP^cre/+^;Sufu^fl/fl^* mice (Figure 1B). The thickness of the VZ/SVZ, defined as the DAPI-dense regions with sparse Tuj1 staining along the lateral ventricles, was significantly increased in the *hGFAP^cre/+^;Sufu^fl/fl^* neocortex compared to controls (Figure 1C). The expansion of VZ/SVZ did not correlate with an increase in the thickness of Pax6+ VZ regions (Figure 1D,E). Rather, we observed an increase in the thickness of Tbr2+ SVZ regions (Figure 1F,G). These findings prompted examination of the proliferative state of the neocortical progenitors in the VZ/SVZ.

### 3.2. Abnormal Proliferation of Cortical Progenitors in the E16.5 Neocortex of hGFAP^cre/+^;Sufu^fl/fl^ Mice

**Figure 2:**
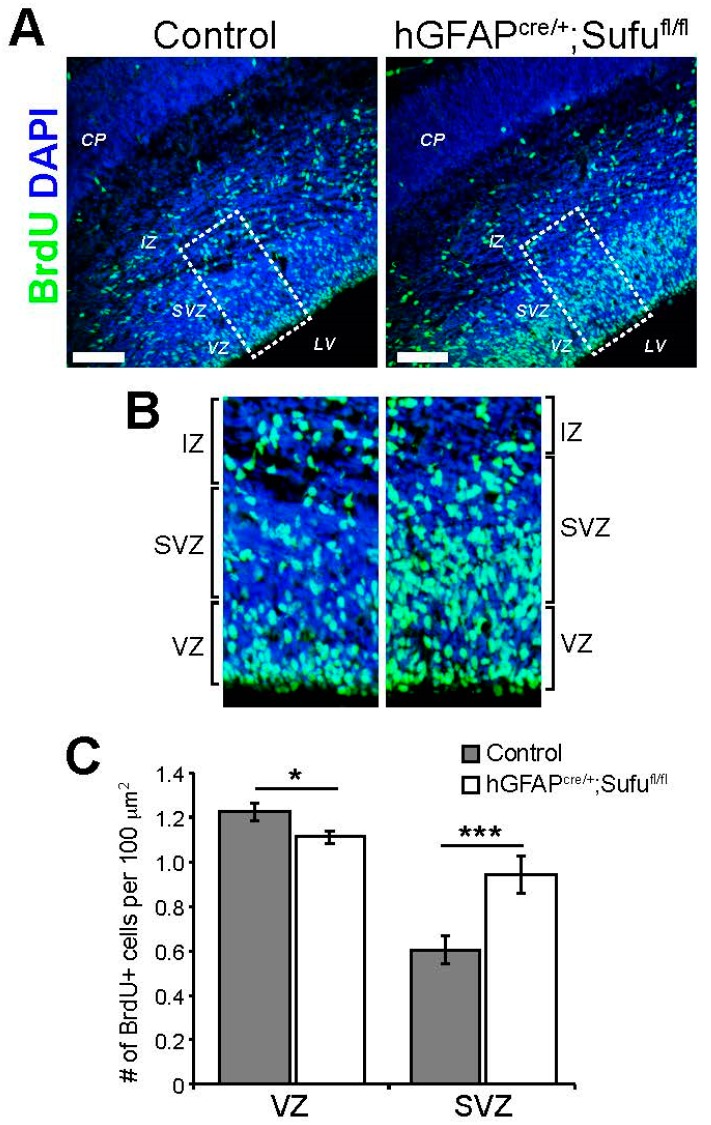
Abnormal proliferation of cortical progenitors in the E16.5 *hGFAP^cre/+^;Sufu^fl/fl^* neocortex; (**A**,**B**) Immunofluorescence staining with anti-BrdU showed that after 4 h of BrdU-treatment, BrdU+ cells were greater in the E16.5 *hGFAP^cre/+^;Sufu^fl/fl^* neocortex compared to control littermates (**A**); Boxed areas in (**A**) showed that this increase was apparent in the SVZ of the E16.5 *hGFAP^cre/+^;Sufu^fl/fl^* neocortex (**B**); Scale bars = 100 μm; CP, cortical plate; BrdU, 5-bromo-2-deoxyuridine (**C**) Quantification of BrdU+ cells within the VZ were mildly, yet significantly reduced, in the E16.5 *hGFAP^cre/+^;Sufu^fl/fl^* neocortex. In contrast, a significant increase in the density of BrdU+ cells were quantified in the SVZ of the E16.5 *hGFAP^cre/+^;Sufu^fl/fl^* neocortex compared to controls; * *p*-value < 0.05, *** *p*-value < 0.01.

At E16.5, the VZ and SVZ are composed of RG and IP cells, respectively [21]. The embryonic neocortex has accumulated a larger population of IPs at this stage as a result of RG cell expansion at earlier stages of corticogenesis. IP cells will undergo one or more cell divisions prior to differentiating into projection neurons that will eventually populate the upper cortical layers [2,4]. To determine whether the expansion of VZ/SVZ in the E16.5 *hGFAP^cre/+^;Sufu^fl/fl^* neocortex was due to deregulated proliferation of cortical progenitors, we conducted BrdU treatments to label proliferating cells. Examination of BrdU-treated embryos 4 h after treatment showed a visible increase in BrdU-labeled cells in the SVZ of the E16.5 *hGFAP^cre/+^;Sufu^fl/fl^* neocortex compared to control (Figure 2A,B). Quantification of BrdU-labeled cells verified these observations in which a significantly higher density of BrdU+ cells was evident in the SVZ of the E16.5 neocortex of *hGFAP^cre/+^;Sufu^fl/fl^* mice, while a slight reduction in BrdU+ cells was observed in the VZ when compared to controls (Figure 2C). These findings indicated that a larger proportion of progenitors were actively dividing in this period in the *hGFAP^cre/+^;Sufu^fl/fl^* neocortical SVZ.

### 3.3. Increase in Basally Dividing Progenitors in the E16.5 hGFAP^cre/+^;Sufu^fl/fl^ Neocortex

**Figure 3:**
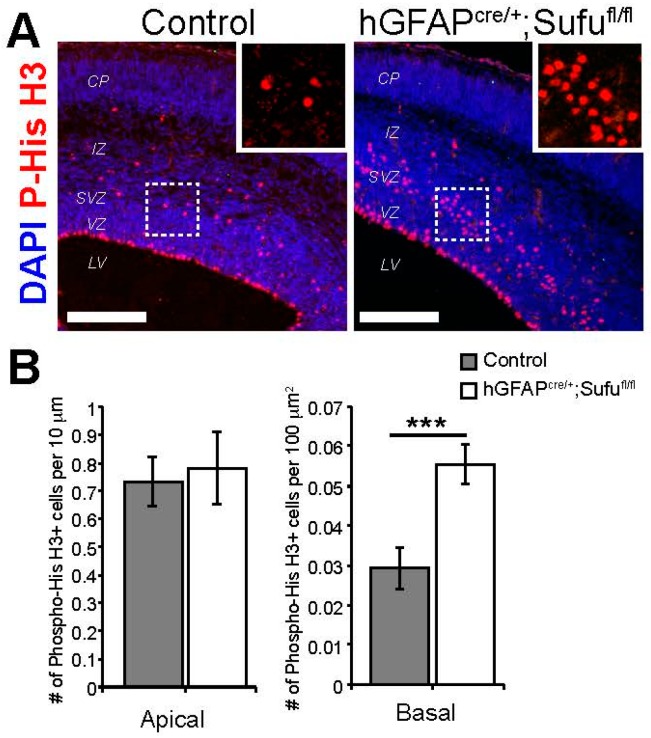
Basally dividing progenitors dramatically increased in the neocortex of E16.5 *hGFAP^cre/+^;Sufu^fl/fl^* mice. (**A**) Labeling of mitotically active cells by immunofluorescence staining against Phospho-Histone H3 (P-His H3) showed an obvious expansion of cells undergoing mitosis in the SVZ of the *hGFAP^cre/+^;Sufu^fl/fl^* neocortex compared to control littermates (boxed inset). Scale bars = 200 μm; (**B**) Quantification of mitotically active cells along the apical lining of the VZ, as labeled by P-His H3, showed no difference between control and *hGFAP^cre/+^;Sufu^fl/fl^* mice. However, a significant almost two-fold increase in P-His H3+ cells were quantified in the SVZ of the *hGFAP^cre/+^;Sufu^fl/fl^* neocortex compared to controls; *** *p*-value < 0.01.

RGs are typically undergoing asymmetric cell division in the apical lining of the VZ, while IPs are undergoing symmetric division in the basal SVZ regions, prior to differentiating into upper layer projection neurons mid-corticogenesis [2]. The increase in density of BrdU+ cells in the SVZ of *hGFAP^cre/+^;Sufu^fl/fl^* mice (Figure 2B) indicates a potential increase in basally dividing progenitors. However, because BrdU is incorporated during the S-phase of the cell cycle along the VZ/SVZ, including RGs that may be undergoing interkinetic nuclear migration [1], BrdU-labeled cells in the SVZ may not be exclusively IPs. We therefore conducted immunofluorescence staining against the mitosis marker, Phospho-Histone H3, to distinguish between RGs undergoing mitosis in the apical lining of the VZ, and IPs that undergo mitosis in the basal or SVZ regions of the neocortical progenitor zone. These experiments showed a dramatic increase in basally dividing cells in the E16.5 *hGFAP^cre/+^;Sufu^fl/fl^* neocortex (boxed inset, Figure 3A). Indeed, we quantified a significant increase in basally dividing progenitors in the E16.5 *hGFAP^cre/+^;Sufu^fl/fl^* neocortex but not in apically dividing cells (Figure 3B). These findings indicated that loss of Sufu promoted the mitotic division of progenitors within the SVZ.

**Figure 4:**
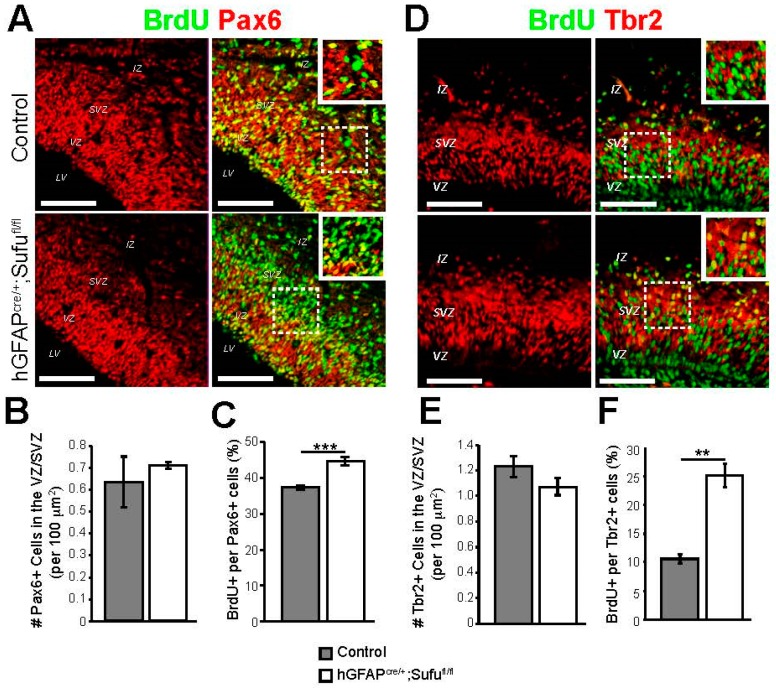
Loss of Sufu led to expansion of Pax6+ and Tbr2+ basally dividing cells. (**A**–**C**) Pax6 and BrdU double immunostaining (**A**) did not show obvious differences in the density of Pax6+ RG cells in the VZ/SVZ between control and *hGFAP^cre/+^;Sufu^fl/fl^* mice at E16.5 and verified by quantification (**B**); On the other hand, BrdU-labeled Pax6+ cells were visibly obvious (boxed insets, **A**) and significantly increased in the E16.5 *hGFAP^cre/+^;Sufu^fl/fl^* neocortex compared to controls (**C**); (**D**–**F**) Tbr2 and BrdU double immunostaining (**D**) did not show obvious differences in the density of Tbr2+ IP cells in the VZ/SVZ between control and *hGFAP^cre/+^;Sufu^fl/fl^* mice at E16.5. This was verified by quantification of Tbr2+ cell density (**E**); However, a greater number of BrdU-labeled Tbr2+ cells was observed within the VZ/SVZ of *hGFAP^cre/+^;Sufu^fl/fl^* mice at E16.5 (boxed inset, **D**); Quantification of double-labeled IP cells was measured and yielded a significantly higher percentage of BrdU-labeled Tbr2+ cells in the E16.5 *hGFAP^cre/+^;Sufu^fl/fl^* VZ/SVZ compared to control littermates (**F**); ** *p*-value < 0.03; *** *p*-value < 0.01. Scale bars = 200 μm; LV, lateral ventricle.

To determine the identity of proliferating cells in the VZ/SVZ, we conducted double immunofluorescence staining with antibodies against BrdU and Pax6, a marker for both apical RG cells and basal RG cells (those that localize in the SVZ), or Tbr2, a specific marker for IPs [4]. As expected, we found an increase in BrdU-labeled Tbr2+ cells in the VZ/SVZ of the E16.5 *hGFAP^cre/+^;Sufu^fl/fl^* neocortex (Figure 4D,F). Surprisingly, we also found an increase in BrdU-labeled Pax6+ cells (Figure 4C), particularly those that localized in the SVZ (boxed areas in Figure 4A). Despite this, we did not observe any changes in the density of Pax6+ RG cells or Tbr2+ IP cells in the VZ/SVZ regions (Figure 4B,E). Since we observed an expansion of Tbr2+ regions (Figure 1F,G), an increase in basally dividing cells (Figure 3), and that Pax6 expression is typically found in newly generated IP cells [21], our findings suggested that loss of Sufu not only promoted the proliferation of basally dividing progenitors, but also likely triggered the transition of Pax6+ RG cells into Tbr2+ IP cells, in the E16.5 neocortex.

### 3.4. Reduced Levels of Gli3R in the E16.5 hGFAP^cre/+^;Sufu^fl/fl^ Neocortex

**Figure 5:**
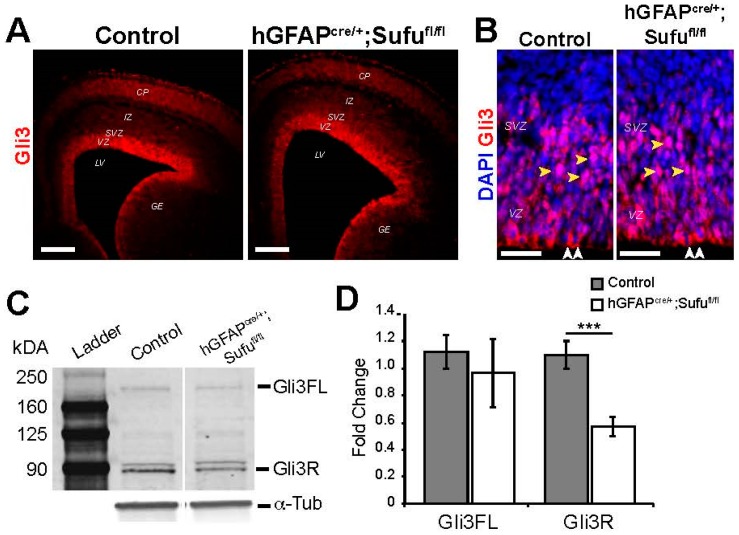
Reduced Gli3R levels in the VZ/SVZ of the E16.5 *hGFAP^cre/+^;Sufu^fl/fl^* neocortex. (**A**,**B**) Gli3 immunostaining showed highly specific expression in the VZ/SVZ of the E16.5 neocortex of control and *hGFAP^cre/+^;Sufu^fl/fl^* mice (**A**); Confocal z-stack imaging showed that the subcellular localization of Gli3 did not significantly differ between control and *hGFAP^cre/+^;Sufu^fl/fl^* VZ/SVZ, where Gli3 appeared to be cytoplasmic along the ventricular lining (white arrows) and nuclear localized in the SVZ (yellow arrows) (**B**); Scale bars = 200 μm in (**A**); 50 μm in (**B**); GE, ganglionic eminence; (**C**) Western blot analysis of protein extracts from the E16.5 neocortex showed the presence of both Gli3FL and cleaved Gli3R proteins, with Gli3R being predominant in both control and *hGFAP^cre/+^;Sufu^fl/fl^* mice. However, quantification of Gli3FL and Gli3R (**D**) showed significantly reduced Gli3R protein levels in the *hGFAP^cre/+^;Sufu^fl/fl^* neocortex compared to controls. α-Tubulin (α-Tub) was used as a loading control; *** *p*-value < 0.01; Gli3FL, full-length Gli3; Gli3R, cleaved Gli3R repressor.

Deletion of Sufu in the mid-corticogenesis stage does not affect the stability of total Gli3 protein because both Gli3FL and its cleaved Gli3R isoform were detectable in the E16.5 *hGFAP^cre/+^;Sufu^fl/fl^* neocortex [6]. We conducted immunostaining against Gli3 in the E16.5 control and *hGFAP^cre/+^;Sufu^fl/fl^* neocortex and found that Gli3 was indeed expressed in the progenitor zones of mice from both genotypes (Figure 5A). Examination of the subcellular localization of Gli3 showed that in both control and mutant neocortex, Gli3 was largely cytoplasmic along the VZ lining (white arrows, Figure 5B) but usually localized in the nucleus as cells transition into the SVZ (yellow arrows, Figure 5B). Western blot analysis of Gli3 proteins showed that both Gli3FL and Gli3R were present in the E16.5 *hGFAP^cre/+^;Sufu^fl/fl^* neocortex, as we previously observed (Figure 5B) [6]. However, quantification of these isoforms showed that Gli3R protein levels were significantly reduced in the E16.5 *hGFAP^cre/+^;Sufu^fl/fl^* neocortex. These findings indicated that in the mid-corticogenesis stage, Sufu does not affect the stability of Gli3FL, nor did it affect the subcellular localization of Gli3R, the predominant form of Gli3 in the embryonic neocortex [7]. Rather, loss of Sufu mid-corticogenesis resulted in the failure to maintain levels of Gli3R in the progenitor zones of the E16.5 *hGFAP^cre/+^;Sufu^fl/fl^* neocortex.

### 3.5. Activation of Shh Signaling in the E16.5 hGFAP^cre/+^;Sufu^fl/fl^ Neocortex

**Figure 6:**
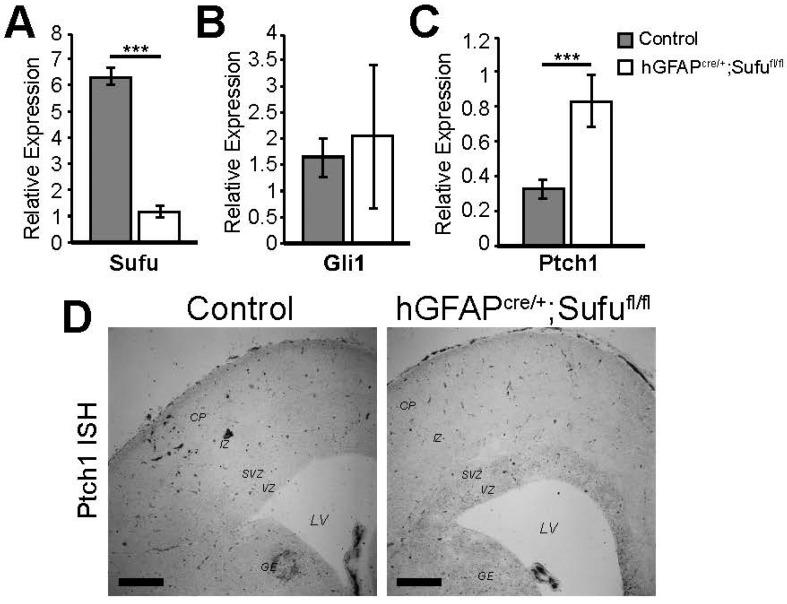
Ectopic expression of Ptch1 in the E16.5 *hGFAP^cre/+^;Sufu^fl/fl^* VZ/SVZ. (**A**–**C**) quantitative PCR (qPCR) analysis of mRNA extracted from the neocortex showed downregulation of Sufu as expected in the E16.5 *hGFAP^cre/+^;Sufu^fl/fl^* neocortex (**A**); No changes in Gli1 expression were detected between control and mutant neocortex as previously reported (**B**); However, a significant increase in the expression of Ptch1 was detected in the E16.5 *hGFAP^cre/+^;Sufu^fl/fl^* neocortex compared to controls (**C**) indicating activation of Sonic Hedgehog (Shh) signaling; *** *p*-value < 0.01; (**D**) In situ hybridization using Patched-1 (Ptch1)-specific riboprobes showed ectopic expression of Ptch1 in the VZ/SVZ of the E16.5 *hGFAP^cre/+^;Sufu^fl/fl^* neocortex. Scale bars = 200 μm.

Since Gli3R is known to inhibit transcription of Shh targets, we examined the activity of Shh signaling in the E16.5 *hGFAP^cre/+^;Sufu^fl/fl^* neocortex. We previously did not observe ectopic Gli1 expression in the E16.5 *hGFAP^cre/+^;Sufu^fl/fl^* neocortex in mice that also carried the Shh-responsive Gli1-LacZ transgene [6,22]. Because the responsiveness of Gli1-LacZ might be tissue- and age- specific, we examined the expression of another Shh target, Ptch1, in the E16.5 neocortex of control and mutant mice by conducting qPCR. As a control, we conducted qPCR using Sufu-specific probes, which verified reduced mRNA expression levels of Sufu in the E16.5 *hGFAP^cre/+^;Sufu^fl/fl^* neocortex (Figure 6A). We also confirmed our previous observation that Gli1 was not ectopically expressed in the E16.5 *hGFAP^cre/+^;Sufu^fl/fl^* neocortex (Figure 6B). However, a significant increase in Ptch1 expression was observed in the E16.5 *hGFAP^cre/+^;Sufu^fl/fl^* neocortex compared to controls (Figure 6C). This was verified by in situ hybridization using Ptch1-specific riboprobes showing ectopic expression of Ptch1 in the VZ/SVZ of the E16.5 *hGFAP^cre/+^;Sufu^fl/fl^* neocortex (Figure 6D). These findings indicated that loss of Sufu resulted in the upregulation of the Shh target Ptch1, a potential consequence of reduced Gli3R levels. Thus, in the E16.5 neocortex, Sufu continues to function as a modulator of Shh signaling in cortical progenitors within the neurogenic zones.

### 3.6. Increase in Cell Death in the E16.5 hGFAP^cre/+^;Sufu^fl/fl^ Neocortex

**Figure 7:**
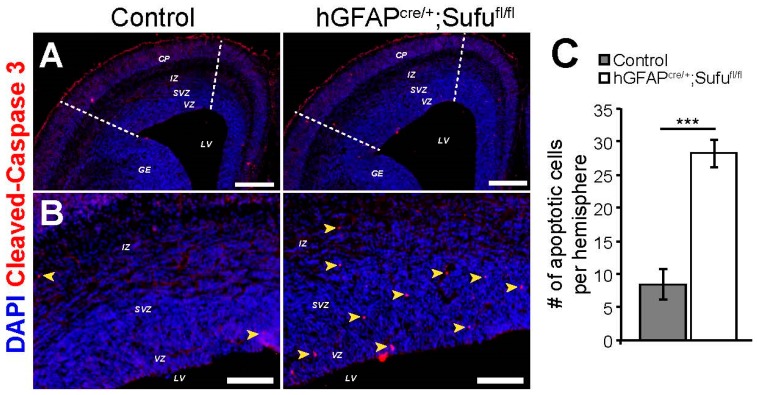
Increased cell death in the E16.5 *hGFAP^cre/+^;Sufu^fl/fl^* dorsolateral neocortex. (**A**,**B**) Immunostaining against the cell death marker, cleaved-Caspase 3 showed an increase in apoptotic cells within the dorsolateral neocortex (defined by dashed lines in **A**); and as marked by yellow arrows in high magnification images (**B**); in the E16.5 *hGFAP^cre/+^;Sufu^fl/fl^* neocortex. Scale bars = 500 μm (**A**); 100 μm (**B**); (**C**) Quantification of cleaved-Caspase 3–positive cells in the dorsolateral region of the neocortical hemispheres confirmed the significant and dramatic increase in apoptotic cells in the E16.5 *hGFAP^cre/+^;Sufu^fl/fl^* neocortex compared to controls; *** *p*-value < 0.01.

We previously observed that loss of Sufu at early stages of corticogenesis resulted in cortical progenitors or neurons that were unable to survive [6]. Given that there is a significant increase in IP cells in the E16.5 *hGFAP^cre/+^;Sufu^fl/fl^* neocortex, we examined whether these progenitors and their progenies were able to survive in the developing neocortex. By immunolabeling with the cell death marker, cleaved-Caspase 3, we found that there was a dramatic increase in apoptotic cells in the dorsolateral neocortex of the E16.5 *hGFAP^cre/+^;Sufu^fl/fl^* mice (within dashed lines, Figure 7A) compared to controls, particularly in the VZ/SVZ regions (Figure 7B). Quantification of cleaved-Caspase 3+ cells confirmed these observations, in which a significantly higher number of apoptotic cells were found in the dorsolateral neocortex of the E16.5 *hGFAP^cre/+^;Sufu^fl/fl^* mice compared to controls (Figure 7C). These findings indicated that the deletion of Sufu in cortical progenitors affected its ability to survive during corticogenesis.

### 3.7. Increase in the Number of Cux1+ Upper Layer Neurons in the P7 hGFAP^cre/+^;Sufu^fl/fl^ Neocortex

**Figure 8:**
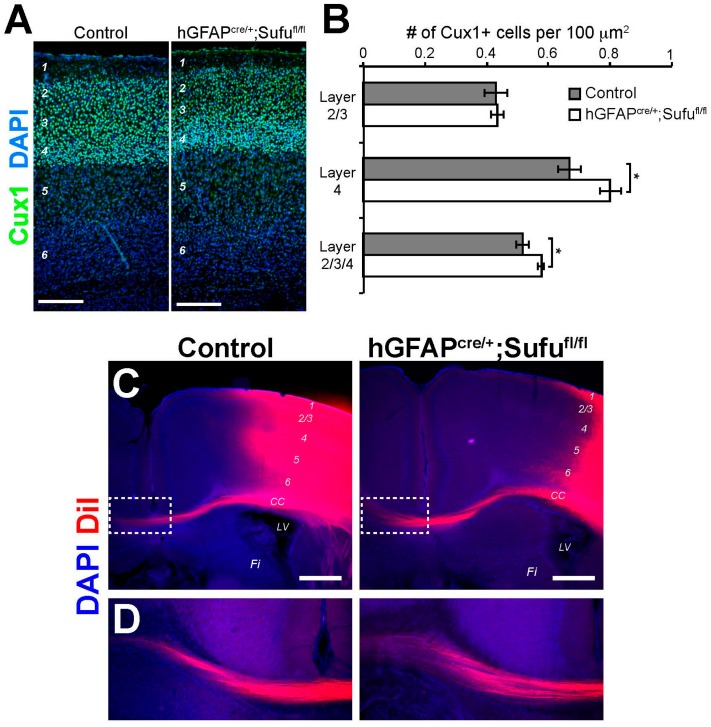
Mild increase in Cux1+ projection neurons and normal callosal projections in the postnatal *hGFAP^cre/+^;Sufu^fl/fl^* neocortex. (**A**,**B**) Cux1 immunolabeling of upper layer projection neurons showed a comparable distribution of Cux1+ neurons, with a densely packed layer 4, in the P7 *hGFAP^cre/+^;Sufu^fl/fl^* neocortex (**A**); Quantification of Cux1+ projection neurons (**B**) yielded comparable densities of Cux1+ neurons in Layer 2/3 between control and *hGFAP^cre/+^;Sufu^fl/fl^* neocortex. A borderline increase in the density of Cux1+ neurons were quantified in layers 2–4 of the P7 *hGFAP^cre/+^;Sufu^fl/fl^* neocortex (*p*-value = 0.054) and appears to be due to a mild increase in the density of Cux1+ neurons in layer 4 (*p*-value = 0.058). Scale bars = 200 μm. CC, corpus callosum; LV, lateral ventricles; FI, fimbria; (**C**,**D**) DiI tracing showed that callosal projections that originate from upper cortical layers were grossly unaffected in the P15 *hGFAP^cre/+^;Sufu^fl/fl^* neocortex and is comparable with control littermates (**C**); Callosal projections were able to project across the midline into the contralateral hemisphere (boxed, **D**); Scale bars = 500 μm. DiI, 1,1′-dioctadecyl-3,3,3′,3′-tetramethylindocarbocyanine.

At E16.5, RGs and IPs largely generate projection neurons that eventually populate upper layers 2–4 of the postnatal neocortex. We wondered whether the expansion of IPs at E16.5 resulted in an increase in the number of upper layer neurons in the postnatal *hGFAP^cre/+^;Sufu^fl/fl^* neocortex. To test this, we conducted immunofluorescence staining against Cux1, a marker for upper neocortical layers as observed in the control brains. Cux1+ neurons in the P7 *hGFAP^cre/+^;Sufu^fl/fl^* neocortex were properly localized as with controls (Figure 8A). However, we detected a modest increase in the density of Cux1+ neurons in layers 2–4, particularly in layer 4, of the P7 *hGFAP^cre/+^;Sufu^fl/fl^* neocortex compared to controls (Figure 8B). Thus, despite the increase in cell death at E16.5 (Figure 7), the expansion of basally-dividing progenitors in the E16.5 *hGFAP^cre/+^;Sufu^fl/fl^* neocortex still resulted in an increase in Cux1+ projection neurons. These observations also verified that the differentiation capacity of surviving cortical progenitors in the E16.5 *hGFAP^cre/+^;Sufu^fl/fl^* neocortex were not compromised. These Cux1+ projection neurons were able to migrate out of the progenitor zones and setlle into the upper cortical layers, particularly in layer 4, of the postnatal *hGFAP^cre/+^;Sufu^fl/fl^* neocortex.

### 3.8. Normal Formation of Callosal Projections in the P15 hGFAP^cre/+^;Sufu^fl/fl^ Neocortex

Cux1+ projection neurons of the upper layers of the neocortex form callosal projections, which interconnect the two cortical hemispheres via the corpus callosum [23]. To examine whether callosal projections were affected in the *hGFAP^cre/+^;Sufu^fl/fl^* neocortex as a consequence of the increase in Cux1+ cells, we embedded DiI crystals in the upper cortical layers of the P15 control and mutant mice to anterogradely label axons crossing the midline along the corpus callosum. As with the controls, we found that DiI-labeled projections in the *hGFAP^cre/+^;Sufu^fl/fl^* neocortex were present in the corpus callosum (Figure 8C) and were able to cross the midline (Figure 8D). These findings indicated that callosal projections typically formed by Cux1+ upper layer neurons were properly generated and projected into the contralateral hemisphere of the *hGFAP^cre/+^;Sufu^fl/fl^* neocortex similar to control littermates.

## 4. Discussion

During neocortical development, the proliferation and differentiation of neural progenitors are tightly controlled to generate predetermined numbers of specific projection neuron subtypes [24]. Although key developmental signaling pathways are known to regulate these processes, modulators of these signaling events have yet to be identified and characterized, particularly those that exert fine control over the behavior of specific progenitor populations. In this study, we provide evidence that the cytoplasmic protein, Sufu, plays a significant role in controlling IP cell number in the progenitor zones of the embryonic neocortex, possibly through its antagonistic role on the Shh signaling pathway.

Our studies show that loss of Sufu specifically affects the generation and proliferation of IPs. At E16.5, most IP cells generate Cux1+ projection neurons that migrate and settle into cortical layers 2–4 of the postnatal neocortex. In the *hGFAP^cre/+^;Sufu^fl/fl^* embryonic neocortex, Sufu deletion led to the expansion of Tbr2+ IP cells in the SVZ. These observations are consistent with previous studies, where elevated or deregulated Shh signaling resulted in the expansion of basal radial glial cells and Tbr2+ IP cells, leading to changes in the mouse neocortex that resemble processes underlying gyrification in the human brain [25]. Our findings are also in line with previous findings by Shikata et al. where the upregulation of Shh signaling enhanced the transition of RG progenitors into IPs [8]. The increase in IP cells contributed to the expansion of VZ/SVZ regions of the E16.5 *hGFAP^cre/+^;Sufu^fl/fl^* neocortex. Therefore, our studies pinpoint a specific role for Sufu in regulating the cell cycle dynamics of RG and IP cells during mid-corticogenesis.

Shh signaling is essential in the developing forebrain, with established roles in axis formation and maintenance of cortical interneuron progenitor identity [5]. In the dorsal forebrain, Shh activity is maintained at low levels since an increase in Shh activity, particularly at early stages of corticogenesis, leads to severe malformation of the neocortex [6,26]. However, in the mid-corticogenesis stage (E14.5 to E17.5), there are more sources of Shh ligands in the neocortex where it functions as a mitogen for cortical progenitors and is subject to modulation by known Shh antagonists [27]. Our findings show that Sufu continues to be an important modulator of Shh activity in the neocortex during mid-corticogenesis. This provides another level at which Shh signaling is tightly regulated by Sufu during cortical development.

Regulating the stability, localization, and transcriptional activity of Gli transcription factors are the primary modes by which Sufu exerts its antagonistic function on Shh signaling [10,13,28,29,30,31]. During corticogenesis, the repressor activity of Gli3 is predominant [7]. Our studies showed that although both Gli3FL and Gli3R were generated in the *hGFAP^cre/+^;Sufu^fl/fl^* neocortex at later stages of corticogenesis, a reduction in Gli3R levels were apparent in the VZ/SVZ. We did not observe any changes in Gli3FL levels suggesting that the stability of full-length Gli3 was not compromised in the absence of Sufu, in contrast to its role in early corticogenesis [6]. Additionally, we also did not observe differences in the nuclear localization of Gli3 in control and mutant mice. Thus, Sufu primarily functions to maintain Gli3R levels, perhaps by preventing its degradation [13,29,32] during mid-corticogenesis.

The reduction in Gli3R levels in the E16.5 *hGFAP^cre/+^;Sufu^fl/fl^* neocortex correlated with an increase in the proliferation of IP cells, which subsequently resulted in the expansion of Cux1+ upper layer neurons. These findings are contrary to the effect of completely abolishing Gli3 early in corticogenesis when there were fewer Cux1+ upper layer neurons because IPs exited the cell cycle prematurely [7]. This discrepancy is likely due to the timing of Gli3 ablation. Furthermore, our findings are consistent with the growing evidence that neural progenitors elicit differential responses to Gli3R or Shh signaling in a dose-dependent manner [33,34]. It would be interesting to determine whether reduced Gli3R levels (i.e., in Gli3 heterozygous knockout mice) during mid-corticogenesis similarly result in the expansion of IP cells. Based on our results, we postulate that the effect of Shh signaling in neural progenitor cells in the embryonic neocortex is crucially dependent on the appropriate Gli3R protein levels that must be maintained by Sufu throughout corticogenesis. However, we also cannot exclude the possibility that Sufu regulates additional signaling pathways, besides its regulatory role in Gli3, to control the behavior of cortical progenitors. Indeed, Sufu has been shown to regulate components of Wnt signaling [35,36], another important regulator of progenitor proliferation and differentiation in the neocortex [37].

In the postnatal *hGFAP^cre/+^;Sufu^fl/fl^* neocortex, we found only a modest increase of Cux1+ projection neurons. This modest increase is likely the result of the dramatic increase in apoptotic cells in the E16.5 *hGFAP^cre/+^;Sufu^fl/fl^* neocortex. Although it is possible that Sufu directly regulates cell death signaling pathways, it is also likely that the limited amounts of growth factors within the VZ/SVZ are unable to sustain the dramatic increase in IPs in the VZ/SVZ. Nevertheless, a significant proportion of progenitors were able to differentiate particularly into Cux1+ layer 4 projection neurons. This hints at the possibility that Sufu specifically affects a subset of Tbr2+ cortical progenitors that are destined to become layer 4 projection neurons. Indeed, recent studies have shown that upper layer neurons originate from distinct IP subpopulations [38,39]. Future studies should take into consideration that distinct subsets of IPs and their neuronal progenies may be differentially regulated by molecular factors, such as Sufu.

Investigating the functional consequences of an increase in projection neurons in the neocortex could prove useful in a number of neurodevelopmental disorders, such as autism, in which alterations in neuronal numbers have been observed [40,41]. Our gross analysis of callosal axons showed no obvious defects in the ability of Cux1+ neurons to form these projections. However, we cannot rule out the possibility that callosal projections in the *hGFAP^cre/+^;Sufu^fl/fl^* neocortex inappropriately target neurons in the contralateral hemisphere, disrupt local connections, or exhibit electrophysiological deficits. Furthermore, Cux1+ upper layer neurons represent several subpopulations of projection neurons in the neocortex [23]. Overall, in-depth analysis is necessary to determine how altered production of Cux1+ neurons during corticogenesis, due to Sufu dysfunction and deregulated Shh signaling, affect neuronal connectivity, function, and behavior. These studies could provide a better understanding and diagnosis of neurological abnormalities that relate to abnormal callosal projection neuron development and function.
